# Diagnosis, treatment, and long-term follow-up of a 13-year-old boy with a testicular mixed germ cell tumor (yolk sac tumor and embryonal carcinoma): a case report and literature review

**DOI:** 10.3389/fped.2026.1793767

**Published:** 2026-03-17

**Authors:** Jing Yang, Jianjun Lai, Shiping Zheng, Huanmei Tang

**Affiliations:** 1 Department of Surgery Outpatient, Shandong Provincial Hospital Affiliated to Shandong First Medical University, Jinan, China; 2 Pediatric Surgery, Second Department of Urology, The People’s Hospital of Shouguang, Shouguang, Shandong, China; 3 Supervision Group of Nursing Department, Shandong Provincial Hospital Affiliated to Shandong First Medical University, Jinan, China

**Keywords:** adolescents, early puberty, mixed germ cell tumors, non-seminomatous germ cell tumors, testicular tumors

## Abstract

Testicular germ cell tumors (TGCTs) are rare in children under 15 years of age. We report the first documented case of a mixed yolk sac tumor and embryonal carcinoma in the left testis of a 13-year-old boy in the early stages of puberty (12–14 years). The patient presented with a left scrotal mass, markedly elevated serum alpha-fetoprotein and human chorionic gonadotropin levels, and ultrasonographic findings suggestive of a testicular tumor. Pathological confirmation was obtained after radical orchiectomy, with no recurrence or metastasis observed during 29 months of follow-up. This case report reviews the literature on histologically similar testicular tumors and summarizes diagnostic and management experiences. For testicular mixed germ cell tumors in early adolescence, immediate radical orchiectomy can achieve clinical cure, and long-term follow-up is crucial.

## 1 Introduction

According to the 2022 World Health Organization (WHO) classification, testicular germ cell tumors (TGCTs) are categorized into two distinct entities: tumors originating from germ cell neoplasia *in situ* (GCNIS) and non-GCNIS-derived tumors (2). Non-GCNIS tumors typically occur in prepubertal patients, with a peak incidence at 0–4 years (3). Of these, 60%–75% are benign (primarily teratomas), while yolk sac tumors (YSTs) represent the predominant malignant form (4). In contrast, GCNIS-derived tumors mainly occur in postpubertal patients, with a peak incidence at 15–19 years (3). Among these, approximately 75% are malignant (5), with mixed germ cell tumors (GCTs) being the most frequent histological subtype. GCNIS-derived tumors comprise seminomas and non-seminomatous germ cell tumors (NSGCTs), which include embryonal carcinoma (EC), choriocarcinoma, postpubertal yolk sac tumors, and teratomas. Testicular mixed germ cell tumors(TMGCTs) refer to tumors comprising two or more of these histological components. An analysis of the Surveillance, Epidemiology, and End Results (SEER) database revealed a median diagnostic age of 18 years among 13- to 19-year-old adolescents, with mixed tumors accounting for 68.2% of cases (6). A literature review confirmed this case to be the first reported NSGCT during the early pubertal transition period (12- to 14-year-olds). This provides valuable data for a demographic where management protocols often overlap between pediatric and adult guidelines.

## 2 Case description

A 13-year-old boy presented with a painless left scrotal mass for over 3 months. Physical examination revealed an enlarged and full left scrotum. A solid, non-tender mass measuring approximately 5 × 4.5 × 5 cm was palpated within, exhibiting slight mobility and ill-defined borders. The surface was smooth, and transillumination was negative; the right testis was unremarkable. His past surgical history included a left indirect inguinal hernia repair at 8 years of age.

Laboratory investigations showed markedly elevated serum tumor markers (STMs): human chorionic gonadotropin (hCG) at 14,937 mIU/mL [7,469 × the upper limit of normal (ULN); reference: 0–2 mIU/mL] and alpha-fetoprotein (AFP) at 399.7 ng/mL (40 × the ULN; reference: 0–10 ng/mL). Carcinoembryonic antigen (CEA) and carbohydrate antigen 19-9 (CA19-9) levels were both within normal ranges, ruling out malignant tumors originating from the gastrointestinal tract, hepatobiliary, or pancreatic systems. Cancer antigen 125 (CA125) levels within normal ranges excluded pelvic inflammatory disease. In the context of an unidentified primary tumor, these findings support a diagnosis of primary testicular cancer.

**Figure 1:**
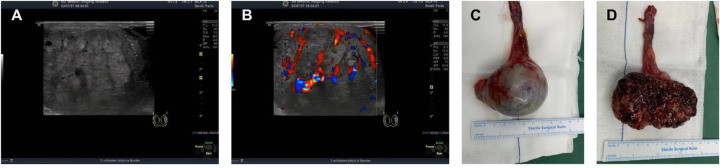
**(A)** Left scrotal ultrasound. **(B)** Color Doppler flow imaging reveals abundant intralesional vascularity with robust blood flow signals. **(C)** An orchiectomy specimen showing a well-encapsulated testicular tumor. **(D)** Macroscopic appearance of the specimen.

Scrotal ultrasonography (US) serves as the primary imaging modality for evaluating painless testicular masses, with a reported sensitivity of 98% and specificity of 95% (7). In this case, ultrasonographic findings (Figures 1A,B) exhibited pathognomonic features of a malignant testicular tumor (8, 9). A morphological evaluation showed that the left testis measured 5.6 × 4.4 × 5.2 cm, containing an irregular, ill-defined hypoechoic mass with heterogeneous echogenicity, internal nodularity, and peripheral punctate calcifications (Figures 1A,B). Color Doppler flow imaging revealed abundant intralesional vascularity (Figure 1B). These ultrasonographic findings indicated a left testicular tumor with marginal calcifications and a grade I left varicocele. No pulmonary metastasis was identified on a chest X-ray. No abdominal or retroperitoneal metastatic lesions were detected on abdominal ultrasound.

**Figure 2:**
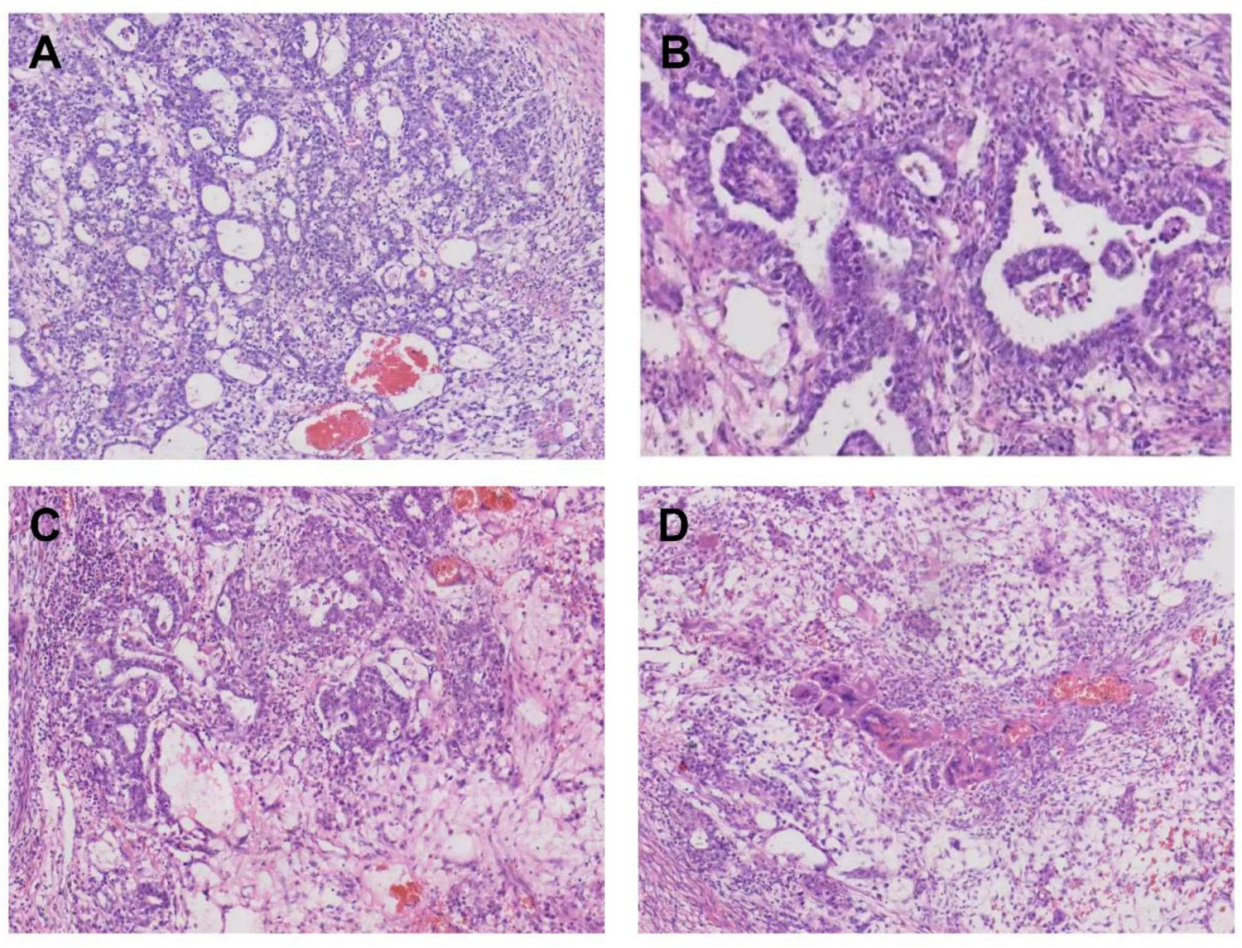
**(A)** Yolk sac tumor region (HE ×200). **(B)** The presence of Schiller–Duvall bodies is characteristic of yolk sac tumors. **(C)** Localized embryonal carcinoma (HE ×200). **(D)** Central syncytiotrophoblastic cells are visible.

A left radical orchiectomy was performed via an inguinal approach (Figures 1C,D). An intraoperative examination confirmed the integrity of the tunica albuginea and spermatic cord structures, with no evidence of tumor invasion. The spermatic cord vessels and vas deferens were ligated and transected at the internal inguinal ring. A gross examination of the resected specimen showed a solid, firm mass with a variegated grayish-white to pale reddish cut surface (Figure 1D). A histopathological analysis confirmed a mixed germ cell tumor consisting of approximately 70% yolk sac tumor and 30% embryonal carcinoma, with focal syncytiotrophoblastic cells (Figure 2). The tumor was confined to the testicular parenchyma, and no lymphovascular invasion was identified.

Immunohistochemical staining supported the pathological diagnosis: the yolk sac tumor component was positive for AFP, GPC3, and SALL4 (10–12); the embryonal carcinoma component stained positively for CD30 and OCT3/4 (10, 13); and syncytiotrophoblastic cells exhibited focal hCG positivity (14), consistent with the markedly elevated preoperative serum hCG level.

At the 1-month postoperative follow-up, the serum tumor markers had normalized: AFP 1.56 ng/mL and hCG <0.2 mIU/mL. Throughout 29 months of systematic follow-up comprising physical examinations, serum tumor marker assays, and scrotal and retroperitoneal ultrasonography, no local recurrence or distant metastasis was identified. The patient demonstrated normal growth and development.

## 3 Discussion

TGCTs represent approximately 0.5% of all malignancies in children under 15 years of age (1), and are exceedingly rare during early puberty. We systematically searched PubMed from 1980 to 2025 using the following keyword combinations: (testicular neoplasms OR testicular tumors) AND (non-seminomatous germ cell tumors OR mixed germ cell tumors) AND (adolescents OR puberty). To our knowledge, this is the first documented case of a testicular mixed germ cell tumor composed of yolk sac tumor and embryonal carcinoma in an early pubertal patient.

YSTs exhibit a bimodal age distribution, with peaks occurring before 4 years and after 15 years of age (15). In prepubertal children, YSTs are the most common malignant testicular tumors and typically present as a pure form with a favorable prognosis (16). In postpubertal patients, however, YSTs are usually mixed variants (12) and exhibit enhanced aggressiveness and a higher recurrence rate (10). EC is a primitive, undifferentiated neoplasm with the capacity to differentiate into various non-seminomatous elements (1). Classified as a postpubertal germ cell tumor (12), EC is the most prevalent malignant TGCT subtype among adolescents (1). Although rare, pure EC is more aggressive than mixed-type EC (12); both pure and dominant EC subtypes are associated with elevated recurrence risks (17, 18). EC and postpubertal YSTs are common components of mixed germ cell tumors (10, 12, 13), and mixed tumors are composed of YST/EC occurring most commonly in patients aged 20–40 years (19–21). Table 1 compares three cases with identical histological components exhibiting markedly different ages at onset and clinical presentations (20, 22, 23).

**Table 1:** Published studies on different age groups with the same pathological type of TGCTs.

Authors, date	Age (year)	Pathology results	Accompanying disease	Treatment	Prognosis
Kuroda et al. (2007) (22)	20 years	>90% EC + YST	Left cryptorchidism and Down syndrome	L: orchiectomy + chemotherapy	NED 7 months after surgery
Mainak et al. (2009) (20)	3 years	EC + YST	Bilateral cryptorchidism	L: orchiectomy R: testicular fixation + chemotherapy	No prognosis
Qin et al. (2017) (23)	38 years	EC predominant + YST	No prognosis	R: orchiectomy + RPLND	NED 3 months after surgery
Present case (2023)	13 years	30% EC + 70% YST	Left oblique hernia	L: orchiectomy	NED 29 months after surgery

EC, embryonic carcinoma; YST, yolk sac tumor; RPLND, retroperitoneal lymph node dissection; NED, no evidence of disease.

Etiological studies of testicular tumors have identified several risk factors, including cryptorchidism and family history (17). Our patient lacked these typical risk factors but had a history of a left indirect inguinal hernia and its surgical repair at age 8. We hypothesize that the sliding hernia sac may have impaired germ cell development by affecting the spermatic cord and scrotum, representing a potential, patient-specific pathogenic mechanism. Supporting this hypothesis, Pottern et al. demonstrated a significantly increased risk of ipsilateral testicular tumors [relative risk (RR) = 4.7, 95% confidence interval (CI): 1.0-21.9] in patients who underwent hernia repair between the ages of 8 and 26 (24). As a congenital anomaly, a pediatric inguinal hernia may elevate scrotal temperature because of the presence of hernial contents in the scrotum and may compress spermatic vessels, potentially resulting in testicular congestion, ischemia, or even necrosis (25). A 2010 meta-analysis indicated that an inguinal hernia is an independent risk factor for testicular cancer [odds ratio (OR) = 1.63, 95% CI: 1.37–1.94, *I*² = 38%], with a lower risk level than cryptorchidism (OR = 4.30, 95% CI: 3.62–5.11, *I*^2^ = 44%) and higher than twinning (OR = 1.22, 95% CI: 1.03–1.44, *I*^2^ = 22%) (26). Furthermore, developmental urogenital anomalies—such as cryptorchidism, hypospadias, and inguinal hernias—are associated with an elevated risk of testicular tumors (27). Notably, the varicocele coexisting in this patient was not associated with testicular tumor development (28). Based on this literature review, further research is still needed to explore the correlation between inguinal hernias and their repair with testicular tumors.

When ultrasonography reveals an intratesticular lesion, serum tumor markers (AFP and hCG) must be measured for diagnostic and therapeutic guidance. AFP and hCG are the most specific and sensitive serum markers for diagnosing testicular tumors (29). In this case, an AFP level of 399.7 ng/mL strongly indicates a yolk sac tumor component, while an elevated hCG level of 14,937 mIU/mL typically suggests choriocarcinoma or syncytiotrophoblastic cell elements. Concurrent elevation of both markers usually suggests a mixed GCT, and their levels exhibit a significant negative correlation with patient age, peaking in adolescents under 20 years old (30). In this case, the markedly elevated AFP level is consistent with adolescent GCT features and corroborates the pathological finding of a 70% yolk sac tumor component. In patients with clinical stage I GCT, hCG levels exhibit a positive correlation with tumor volume (30). In this case, the extreme hCG elevation is strongly associated with both the large tumor size (5.6 × 4.4 × 5.2 cm) and hypersecretion by syncytiotrophoblastic cells within the embryonal carcinoma (Figure 2D).

The clinical presentation, imaging findings, and elevated tumor markers strongly suggested malignancy, prompting a radical orchiectomy. A histopathological analysis confirmed a mixed germ cell tumor (MGCT) composed of 70% yolk sac tumor (YST) and 30% EC. While YST-dominant MGCTs generally exhibit a favorable prognosis owing to platinum sensitivity and low metastatic rates (1), the 30% EC component in this case raises concerns about lymphovascular invasion and potential retroperitoneal or pulmonary metastases. According to the 8th edition of the American Joint Committee on Cancer (AJCC) TNM classification (1), the tumor was staged as pT1N0M0S0, which defines clinical stage I (CSI) MGCT. This staging was defined by testicular confinement (pT1), the absence of nodal or distant metastasis (N0M0), and the normalization of tumor markers postoperatively (S0).

International guidelines recommend that patients with CSI MGCTs avoid adjuvant chemotherapy but adhere to stringent surveillance protocols involving regular tumor marker measurements and imaging evaluations. Despite the 5-year relative survival rate reaching 97.4% in patients under 14 years old with localized testicular tumors (31), recurrence risk remains non-negligible. The Children's Oncology Group (COG) established age ≥12 years at diagnosis[hazard ratio (HR) = 8.87, 95% CI: 2.97–26.5, *p* < 0.001] as the strongest independent predictor of recurrence in CSI TGCT (32), necessitating enhanced surveillance for this adolescent population. Currently, there is no consensus on postorchiectomy management strategies for pediatric and adolescent patients with NSGCTs (33), with ongoing controversies surrounding the optimal duration and frequency of follow-up (4).

The age-dependent pathogenesis of TGCTs necessitates stratified management for pediatric patients. Pediatric TGCTs (in patients <12 years) primarily arise from non-germ cell neoplasia *in situ* (non-GCNIS) and exhibit distinct biological behavior compared with adult tumors (2), warranting a separate assessment of their recurrence patterns. As TGCTs in both adolescents (≥12 years) and adults primarily originate from GCNIS; therefore, CSI management in adolescents can adhere to adult guidelines (33). Studies in adult populations have shown that radical orchiectomy alone cures 72% of patients with CSI NSGCTs (34), whereas active surveillance achieves 5-year overall survival rates of 99%, even including salvage therapy after relapse (35). Notably, the vast majority of recurrences in CSI NSGCT occur within 2 years after orchiectomy (36), establishing this period as critical for surveillance.

STMs, specifically AFP and hCG, are pivotal for tumor staging, recurrence surveillance, and prognostic evaluation in the postoperative management of TGCTs (16). The COG trial AGCT0132 demonstrated that 98% of pediatric and adolescent malignant GCT patients with elevated STMs at diagnosis exhibited recurrent STM elevation upon relapse (37), confirming STMs as highly sensitive markers for early postoperative recurrence detection in this population. In this case, the normalization of both AFP and hCG levels within 1 month postoperatively indicated complete eradication of tumor burden. Any persistent or recurrent elevation of STMs often suggests tumor recurrence, metastasis, and an adverse prognosis.

In this case, the patient's serum tumor markers were significantly elevated prior to surgery but rapidly returned to normal levels postoperatively. An abdominal ultrasound, chest radiography, and intraoperative exploration revealed no evidence of retroperitoneal lymph node or distant metastases, consistent with a clinical stage I testicular tumor. Although CT/MRI remains the gold standard for evaluating retroperitoneal and distant metastases (38), we omitted cross-sectional imaging during initial staging and routine follow-up for this patient with a confirmed stage I tumor, given the radiation hazards of CT, the limited availability of pediatric MRI data (38), and the need to adhere to the ALARA principle in children. Based on COG data from 2015 to 2019 (3, 37), 98% of recurrences can be detected early through serial tumor marker monitoring. We therefore implemented a surveillance protocol combining marker assays, scrotal/retroperitoneal ultrasounds, and physical examinations (4). Surveillance was performed monthly for the first 6 months, quarterly from 7 to 24 months, biannually from 3 to 5 years, and annually thereafter. MRI was reserved for cases with abnormal markers or suspicious ultrasound findings. This protocol is more rigorous than adult guidelines (AUA) (39), but it achieves early recurrence detection through high-frequency marker monitoring and radiation-free ultrasounds. The patient has now achieved 29 months of disease-free survival, confirming the clinical validity of this management strategy.

## 4 Conclusions

Testicular mixed germ cell tumors (yolk sac tumors with embryonal carcinoma) in early adolescence are clinically rare, and markedly elevated preoperative AFP and hCG levels provide crucial diagnostic value. Timely radical orchiectomy offers curative potential. For clinical stage I patients, routine adjuvant chemotherapy is unnecessary, but close surveillance for at least 2 years is mandatory. An extended follow-up beyond 5 years is essential to monitor late recurrence or metastasis. This case suggests that a history of inguinal hernia and its surgical correction may be risk factors for early adolescent testicular tumors. Potential mechanisms, including chronic inflammation, ischemic injury, and mutagenic accumulation, require further investigation.

## Data Availability

The raw data supporting the conclusions of this article will be made available by the authors, without undue reservation.
